# Upper limb vibration prototype with sports and rehabilitation applications: development, evaluation and preliminary study

**DOI:** 10.1049/htl.2016.0069

**Published:** 2017-02-20

**Authors:** Amit Narahar Pujari, Richard D. Neilson, Sumeet S. Aphale, Marco Cardinale

**Affiliations:** 1 Medical Physics and Clinical Engineering, Nottingham University Hospitals, Nottingham NG7 2UH, UK; 2 School of Engineering, College of Physical Sciences, University of Aberdeen, Aberdeen AB24 3FX, UK; 3 Department of Sports Science, Aspire Academy, Doha, Qatar; 4 College of Life Sciences and Medicine, University of Aberdeen, Aberdeen, UK

**Keywords:** sport, gait analysis, patient rehabilitation, electromyography, prototypes, actuators, vibrations, electromyography, neuromuscular stimulation, exercise intervention, rehabilitation, sports, upper limb vibration prototype

## Abstract

Vibration stimulation as an exercise intervention has been studied increasingly for its potential benefits and applications in sports and rehabilitation. Vibratory exercise devices should be capable of generating highly precise and repeatable vibrations and should be capable of generating a range of vibration amplitudes and frequencies in order to provide different training protocols. Many devices used to exercise the upper body provide limited variations to exercise regimes mostly due to the fact that only vibration frequency can be controlled. The authors present an upper limb vibration exercise device with a novel actuator system and design which attempts to address these limitations. Preliminary results show that this device is capable of generating highly precise and repeatable vibrations with independent control over amplitude and frequency. Furthermore, the results also show that this solution provides a higher neuromuscular stimulation (i.e. electromyography activity) when compared with a control condition. The portability of this device is an advantage, and though in its current configuration it may not be suitable for applications requiring higher amplitude levels the technology is scalable.

## 1 Introduction

Recent years have seen a significant increase in studies investigating the use of the vibration stimulation as an exercise intervention. Applications of vibration exercise range from sports to rehabilitation with apparent benefits or improvements in muscle strength, muscle power, body balance, flexibility and bone density [1]. Although the exact physiological mechanisms underlying these benefits are relatively poorly understood, it has been established that vibration stimulation leads to enhanced neuromuscular responses which have been linked to the above-cited benefits [1, 2]. Over the last decade, independent investigators have developed or studied novel vibration exercise devices to be able to enhance muscle strength and bone density [3, 4]. Two main types of vibration devices exists, those which deliver the vibration through the lower limbs and are referred to as whole body vibration (WBV) devices and those which stimulate the muscles of the hand and the arm, referred to as upper limb vibration (ULV) devices. Typically, the WBV devices deliver the vibration to the user's lower limbs while the user stands in a half squat position. ULV devices can consist of vibrating dumbbells and/or vibrating pulleys where the user grabs the vibrating actuator or a cable connected to a vibrator in order to receive the vibrations [5]. Few devices currently commercially available do not require or enable the user to expend any voluntary force when performing the vibration exercise. This is an important limitation as voluntary isometric contraction superimposed on vibration stimulation has been shown to further increase neuromuscular activity in both upper and lower limbs [5–7]. Thus, one way to further improve the effectiveness of the vibration exercise could be represented by the superimposition of isometric exercises on vibration stimulation. One of the major limitations of the existing vibration devices is that the vibration frequency and amplitude cannot be always controlled independently, thus restricting the choice of frequency–amplitude combinations, and therefore vibration magnitude available to the user. Considering that different vibration frequencies and amplitudes have been suggested to evoke different neuromuscular responses [8], an important design feature of a vibration exercise device should reside in the ability of delivering various frequency–amplitude combinations to benefit the users [9]. Moreover, many vibration devices tend to be heavy and not easily portable. The work presented here demonstrates a novel design for an ULV device which attempts to address these limitations. The novelty of the device presented in this Letter lies in its actuator which enables the delivery of highly reliable sinusoidal vibration stimulations in which frequency and amplitude can be controlled independently. In addition, due to its smaller size, and practical design, the device is portable and can be easily attached and removed from the desired platform which can act as a base for the vibration delivery. Equally importantly, the design of the device also enables the user to perform isometric exercise while receiving vibration stimulations. This Letter describes the development, operation and evaluation of this novel ULV device and presents the results of a preliminary study to demonstrate the concept and methodology.

## 2 Prototype development

**Fig. 1:**
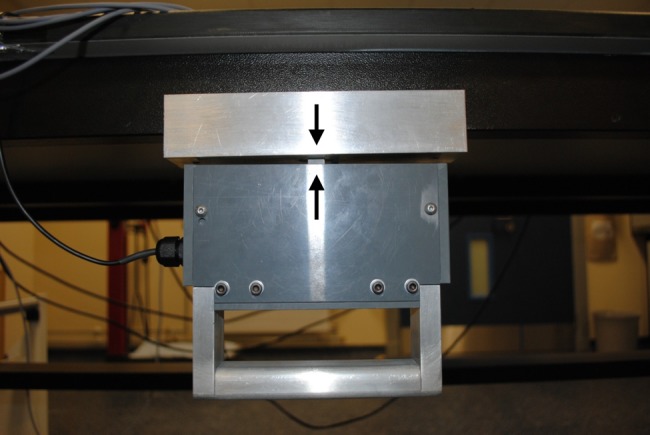
Photograph of the ULV device attached to a side of the table from underneath. The arrows explain the location of the piezoelectric actuator inside the casing; the top and bottom arrow signs on the piezoelectric actuator (Fig. 2) correspond to the upper and lower arrow signs on the casing

**Fig. 2:**
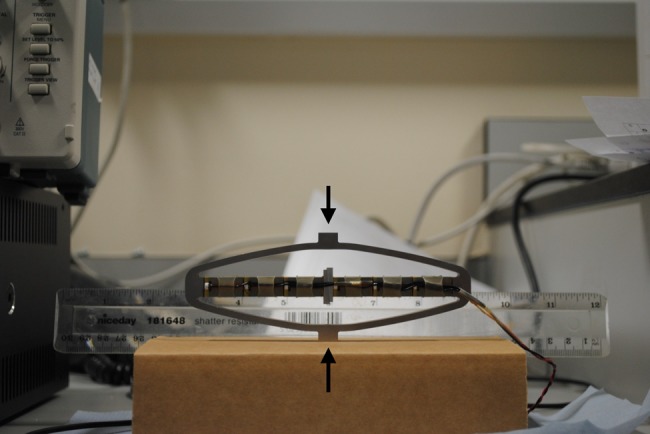
Photograph of the composite, high-displacement APA500L piezoelectric actuator used in this work (as sourced from CEDRAT Technologies), showing its relative scale/size against a ruler

A small-scale ULV device was designed and built (Figs. 1 and 2). The design of the device along with the size of the actuators used as the source of mechanical vibrations ensured that the vibration device is portable.

The actuator assembly used to provide the source of vibration for the neuromuscular stimulation piezoelectric is shown below (Fig. 2). A commercial piezoelectric actuator was chosen because it offered significant advantages in terms of small size, high force and ease of control of the excitation over the other conventional vibration sources.

For Fig. 2, the arrows refer to the top and bottom ends of the actuator. This actuator assembly was fitted inside the electrically insulated casing as shown in Fig. 1.

### 2.1 Prototype description

The ULV device consisted of three principle component groups:

- Vibration device consisting of piezoelectric actuators.
- Amplifier, power source, sensor etc. attached to the vibration device.
- Sensors attached to the body of the user.

**Fig. 3:**
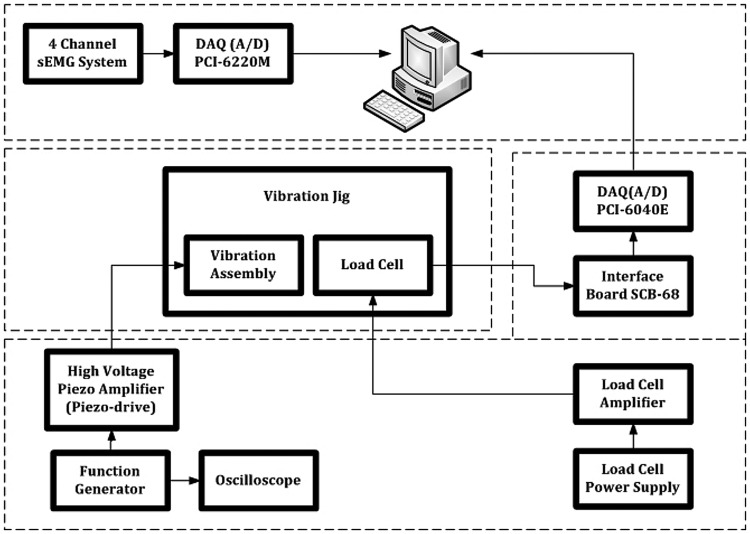
Schematic diagram showing various components of the ULV device and interconnections between those parts

A schematic block-diagram of the complete test prototype of the ULV is shown in Fig. 3.

The top rectangle represents the components from group 3, i.e. the sensors attached to the body of the user. The middle (left) rectangle represents the components of group 1, i.e. the vibration device and the bottom rectangle depicts the components of group 2, i.e. the amplifier, power source etc. The surface electromyography (sEMG) system (i.e. the sensors attached to the body of the user) was connected to the central personal computer (PC) via the data acquisition (DAQ) card (PCI-6220M, National Instruments (NI), TX, USA). The vibration rig was independently powered and operated by a function generator (Type TG302, Levell Instruments, BCC Ltd., UK) and dedicated amplifier (PDX200, PiezoDrive PDX High-Speed Voltage Amplifier, Newcastle Innovation Ltd., Australia). A load cell (LCM703-50, Omega Engineering Inc., UK) housed in the vibration rig was powered independently, though the output of the load cell was fed to the central PC via the DAQ card (PCI-6040E, NI, TX, USA) and an interface board (SCB-68, NI, TX, USA). The following section describes briefly how the device operates.

### 2.2 Operation of the device

**Fig. 4:**
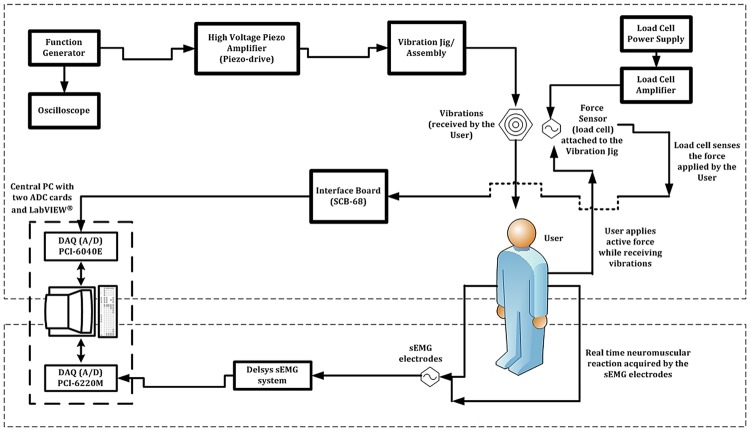
Schematic diagram showing operation of the complete ULV system

The function generator provided the required input waveform for the vibration, please refer to Fig. 4 for the operation of the device. This waveform was delivered to the piezoelectric actuator (APA500L, CEDRAT Technologies, France) through the piezoelectric amplifier.

The characteristics of the input waveform to the piezoelectric were monitored through the oscilloscope (TDS210, Tektronix Inc., OR, USA).

The user experienced the vibration stimulation through the hand–arm while simultaneously delivering an active force against the vibration rig though the device's handle (Figs. 1 and 8). The force applied by the user was sensed continuously by the load cell fitted into the vibration rig. This real-time signal generated by the load cell was transferred back to the central PC through the interface board and DAQ card.

The sEMG system recorded the real-time muscle activity of the user and was connected to the central PC through a separate DAQ card (PCI-6220M, NI, TX, USA) and specialised EMG hardware assembly (Bagnoli 4 channel EMG System with DE-2.1 electrodes, Delsys Inc., MA, USA). The electrodes used were non-invasive and differential to reject the common noise. The user's recorded sEMG signals were acquired and stored on the central PC using dedicated sEMG software (EMGWorks, Delsys Inc., Version 2, MA, USA).

## 3 System performance evaluation

**Fig. 5:**
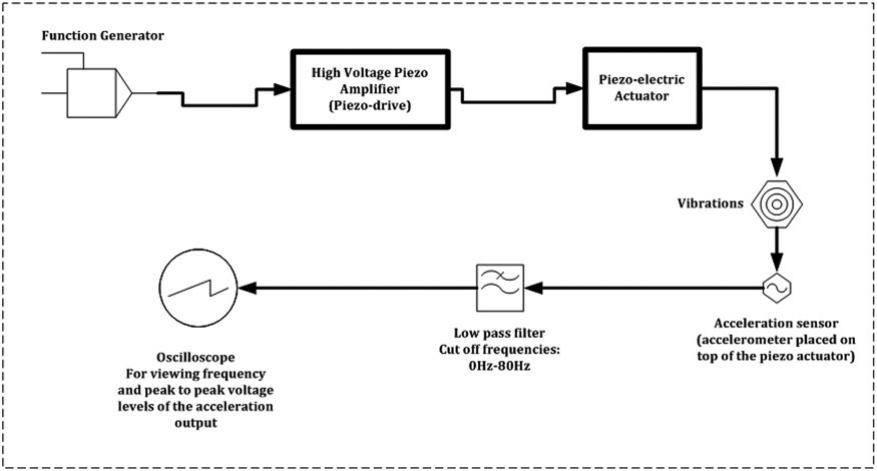
Schematic showing experimental set-up used for recording vibration characteristics to verify and evaluate the performance of the miniature vibration device system

Performance of the ULV (Fig. 5) device was evaluated prior to any experimentation. Performance evaluation was based on the verification of the following points:

- The ability of the miniature vibration system's actuators to generate the required levels of vibration frequencies and amplitudes.
- The levels of input voltages required from the piezoelectric amplifier to produce the desired levels of vibration characteristics.
- The repeatability of generation of the vibration characteristics.
- The reliability and repeatable of the sensor data, e.g. the data from the accelerometer which measures the vibration.

To assess the above requirements, a simple procedure was followed. An input sinusoidal waveform was delivered to the vibration device through the function generator and the amplifier and the corresponding vibration characteristics generated were recorded. This procedure was repeated multiple times to ensure the vibration characteristics values were consistent and hence repeatable. A schematic representation of the experimental set-up used for recording the vibration characteristics is shown in Fig. 5.

The experimental set-up to generate the vibrations was as follows. The vibration characteristics were recorded by placing an accelerometer (ADXL-330, Analog Devices, MA, USA) on the centre top of the bare piezoelectric actuator assembly (corresponding to the top sign in the Fig. 2). The accelerometer signal was recorded, observed and analysed on an oscilloscope. The signal to the oscilloscope from the accelerometer was low-pass filtered with a cut-off frequency 80 Hz. This made sure that the frequency range of interest (30 and 50 Hz) was being recorded and analysed while rejecting any other noise.

## 4 Performance evaluation results

**Fig. 6:**
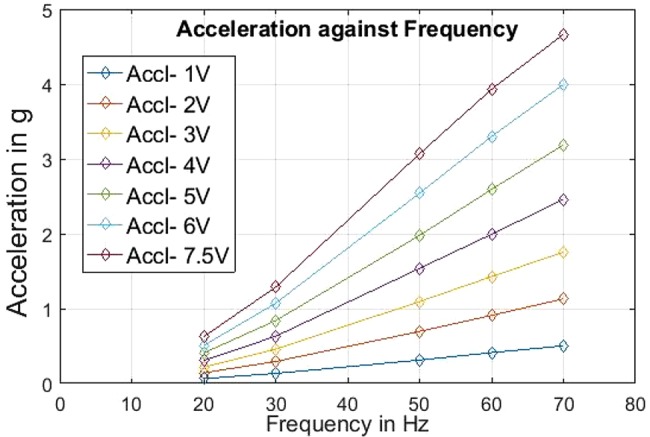
Graph of acceleration levels obtained from the piezoelectric actuator against corresponding vibration frequencies for varying input voltage

**Fig. 7:**
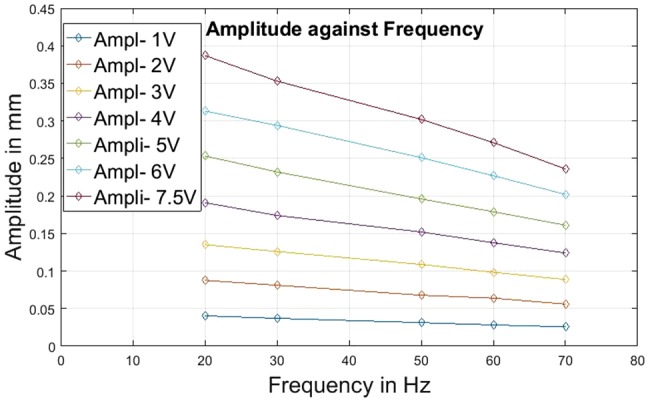
Graph of amplitude levels obtained from the piezoelectric actuator against corresponding vibration frequencies for varying input voltage

One set of complete vibration characteristics’ recording, carried out just before the experiments, is tabulated in Tables 1 and 2 and presented graphically in Figs. 6 and 7. The values in Tables 1 and 2 were used for all the experiments carried out (including preliminary experiments) on the ULV device for this project.

**Fig. 8:**
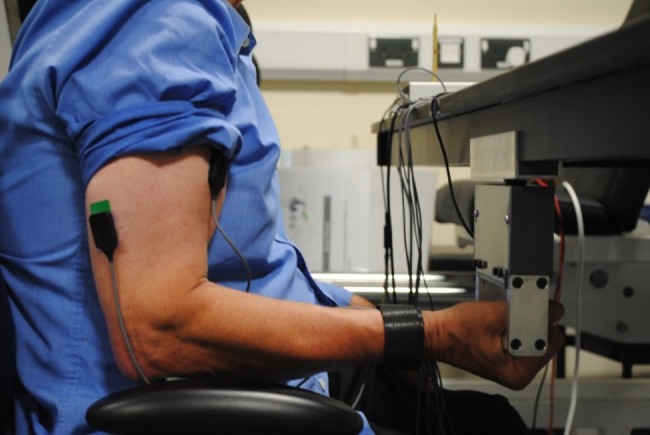
Photograph showing volunteer posture and sensor placement used for the tests

**Table 1:** Acceleration values of piezoelectric actuator against input voltage (V) and frequency (Freq*^n^* in hertz) levels (Accln = acceleration in meter per second square)

Freq*^n^*, Hz	Accl*^n^*, m/s^2^ at (1 V) input	Accl*^n^*, m/s^2^ at (2 V) input	Accl*^n^*, m/s^2^ at (3 V) input	Accl*^n^*, m/s^2^ at (4 V) input	Accl*^n^*, m/s^2^ at (5 V) input	Accl*^n^*, m/s^2^ at (6 V) input	Accl*^n^*, m/s^2^ at (7.5 V) input
20	0.065	0.141	0.217	0.305	0.407	0.506	0.624
30	0.134	0.294	0.458	0.630	0.839	1.071	1.286
50	0.313	0.695	1.094	1.538	1.978	2.542	3.068
60	0.412	0.910	1.428	1.993	2.593	3.300	3.930
70	0.503	1.131	1.756	2.460	3.189	3.995	4.666

**Table 2:** Amplitude values of piezoelectric actuator against input voltage (V) and frequency (Freq*^n^* in hertz) levels (Ampl = amplitude in millimetres)

Freq*^n^*, Hz	Ampl, mm at (1 V) input	Ampl, mm at (2 V) input	Ampl, mm at (3 V) input	Ampl, mm at (4 V) input	Ampl, mm at (5 V) input	Ampl, mm at (6 V) input	Ampl, mm at (7.5 V) input
20	0.040	0.087	0.135	0.191	0.253	0.313	0.387
30	0.037	0.081	0.126	0.174	0.232	0.294	0.353
50	0.031	0.067	0.108	0.152	0.196	0.251	0.302
60	0.028	0.063	0.098	0.137	0.179	0.227	0.271
70	0.025	0.056	0.088	0.124	0.161	0.202	0.236

For the legends of Tables 1 and 2, the voltage (V) was the level of input voltage at function generator applied through sinusoidal signal. The vibration frequency was directly recorded on the oscilloscope, an output from the accelerometer. On the basis of these directly obtained values, the corresponding values of acceleration and amplitudes were calculated using the appropriate calibrations and formulae.

The results (Tables 1 and 2, Figs. 6 and 7) of the performance evaluation confirmed the following observations. The piezoelectric actuator assembly was able to generate the required level of vibration frequencies (20–50 Hz and higher), though the generated amplitudes levels were lower than desired (i.e. <0.5 mm). The corresponding acceleration levels were well within the acceptable and safe level (<5 g) [10]. The amplitude and frequency generation was highly precise and repeatable.

## 5 Preliminary study

After evaluating the performance of the ULV device/system successfully, a small study was carried out to validate the exercise methodology and the utility and effectiveness of such a device to enhance neuromuscular activity.

### 5.1 Participants and study design

About 12 participants [6 males and 6 female, age (28 ± 7.24 years), weight (73.16 ± 11.19 kg) and height (173 ± 13.04 cm)] were recruited and signed an informed written consent. This Letter was approved by the University of Aberdeen's College of Life Sciences and Medicine research ethics committee. In the first visit, each participant was introduced to the ULV device. Then, the participant performed isometric arm flexion exercise of various intensities as a warm up by holding on to the hand grip of the ULV device with elbow flexed at 90° (Fig. 8). Following the warm up, the participants performed three isometric elbow flexion effort of 10 s each at their maximum voluntary contraction (MVC) level using the set-up as shown in Fig. 8.

MVC, the MVC was calculated from the corresponding force values generated by the maximum static load produced by the participant. The maximum static load applied by the participant represented MVC of the targeted muscles. Maximum activation levels of the targeted muscles were obtained from the sEMG activity corresponding to the MVC values of these muscles. That is, the corresponding EMG activity when maximum static load was applied. Each MVC effort was separated by 5 min of rest. During this effort the corresponding force levels and muscle activation (EMG) levels were recorded. An average of the three MVC effort was used to calculate the maximum force level and muscle activation of the individual. In the second and third visits, the exercise treatments consisted of an isometric arm press (flexion) against the ULV device hand-grip with the elbow flexed at 90° for 1 min (Fig. 8). In the second and third visits, participants performed the 1 min elbow flexion on the ULV device at 80% of their MVC capacity under either vibration (V) or control (C = no vibration) conditions each. Thus, participants were randomly allocated either V or C treatment in the second or third visit. The vibration condition (V) was 30 Hz–0.5 mm.

### 5.2 EMG acquisition and data processing

The EMG signal was recorded from the biceps brachii (biceps) and flexor carpi radialis (forearm) muscles using Delsys single differential electrodes (DE-2.1, Delsys Inc., MA, USA). The EMG was sampled at 1000 Hz and analogue filtered at 20–450 Hz by the EMG DAQ system (Bagnoli, Delsys Inc.). Post-processing of the EMG was performed in MATLAB in which a digital notch filter (Butterworth, tenth order, stop band 49.5–50.5) was employed at 50 Hz to attenuate the line interference. The EMG values were normalised with respect to the participants 100% MVC values of the corresponding muscle. Any baseline offset was removed by subtracting the mean before further processing. The 60 s of EMG response from each participant and exercise condition (i.e. both V and C) was divided into five equal sections of 12 s. An average EMG root mean square (RMS) (EMG_rms_) value was derived for each section. The EMG_rms_ values were obtained by using the moving window technique. Initially, the RMS was calculated for each window according to the formula

$${\rm RMS}\left\{{\left\vert {m\lpar t\rpar } \right\vert } \right\}= \left({\displaystyle{1 \over T}\int_t^{t + T} {m^2\lpar t\rpar \, {\rm d}t} } \right)^{1/2}$$

Then, the RMS for the entire data length was obtained by averaging the individual RMS values of each window. The Hamming window was used with a window length of 1 s and no window overlap. Normalisation was performed by dividing the entire section of the data value to be normalised by the maximum value of the sEMG amplitude for that participant for that muscle group. The maximum value was the maximum figure obtained from the MVC effort of the participant under C condition.

### 5.3 Statistical analysis

Alpha was set at 0.05. Paired student *t*-tests (one tail, different variance) were employed to compare EMG responses between C and V conditions and to establish the significance level (*P* value) of the deviations from the means. The distribution of grouped data was assessed for normality using the Lilliefors test with a significance detection level of ≤0.05. Statistical analysis was carried out using the statistical software SigmaPlot (Systat Software Inc., San Jose, CA, Version 12).

## 6 Preliminary study results

**Fig. 9:**
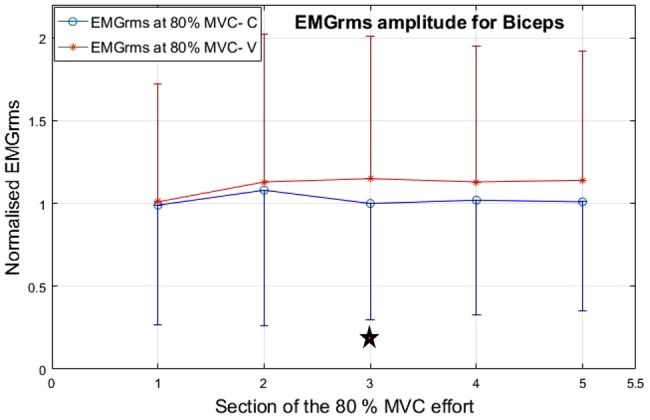
Normalised mean EMG values for the biceps for the five consecutive sections of the isometric contraction effort at 80% of MVC

**Fig. 10:**
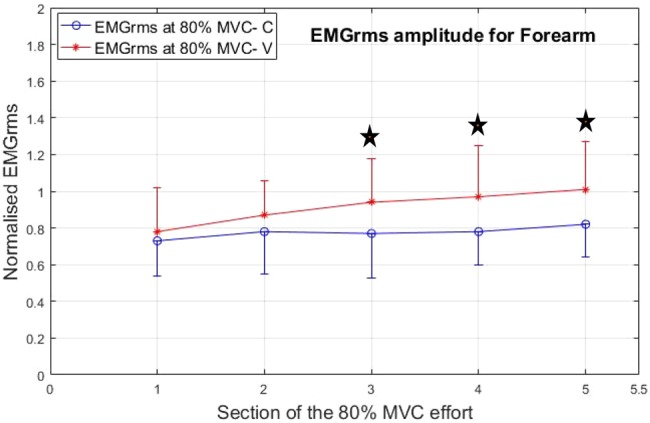
Normalised mean EMG values for the forearm for the five consecutive sections of the isometric contraction effort at 80% of MVC

Both the muscles studied, i.e. the biceps brachii (biceps) and the flexor carpi radialis (forearm) showed higher EMG_rms_ responses under the V condition compared with the C condition (see Figs. 9 and 10 – ‘Star sign’ denotes statistically significant difference between the C and V).

As the exercise/treatment progressed in time, the EMG_rms_ amplitude trend under the V condition separates more from the C condition with the flexor carpi radialis showing the largest difference. The EMG amplitude under the V condition effort increases with a steeper positive slope for both muscle groups, indicating higher neuromuscular activity compared with the corresponding C effort. However, the flexor carpi radialis shows more statistically significantly higher EMG_rms_ amplitude values under the V condition compared with the respective C condition.

## 7 Discussion and conclusions

A novel ULV device capable of delivering highly precise vibration stimulations was developed and evaluated successfully.

Unlike other actuators, the piezoelectric material enables the delivery of highly precise vibration characteristics, in that very small increments in the values of frequency and amplitude can be achieved. This functionality is not available in the current vibration devices. The ability to generate small increments in the frequency and amplitude values increases the range of vibration characteristics that can be employed to induce the highest neuromuscular responses, suitable to participant, their physiological condition and exercise posture and contraction level. The ability to have independent control over frequency and amplitude characteristics extends the utility of this type of device even further. However, this device is not without its limitations. Cost of the piezoelectric material makes such actuators expensive. Furthermore, though the range of frequencies that could generate was well beyond the requirements, the range of vibration amplitude it could generate was limited to maximum 0.5 mm peak-to-peak amplitude. This was limited by piezoelectric crystal's material properties and the design of the actuator. One way to increase the range of amplitude of the actuator would be by stacking the actuators in series, one on top of another. However, this would further increase the cost of the vibration device. Thus, this type of vibration device may not be particularly suitable for generating higher vibration amplitudes (e.g. >1 mm peak to peak) in a cost-effective manner.

The results of the preliminary study show that in the upper limbs, vibration superimposed on isometric contraction induces higher neuromuscular activation compared with isometric contraction alone. This corroborates earlier results indicating isometric contraction superimposed on vibration exercise can enhance neuromuscular response in upper limbs [5]. The Letter demonstrates the utility and effectiveness of such type of ULV stimulation device for the purposes of vibration exercises which could have applications in sports and rehabilitation. However, it is important to note that the main purpose of the above preliminary study was to demonstrate the concept and methodology of this novel device and not to analyse neuromuscular responses in detail. Owing to this reason, the study was limited in scale. Therefore, no overarching conclusions should be drawn. Nevertheless, this study does demonstrate the utility and effectiveness of the device presented here.

## References

[1] Rittweger J.: ‘Vibration as an exercise modality: how it may work, and what its potential might be’, Eur. J. Appl. Physiol., 2010, 108, (5), pp. 877–904 (doi: 10.1007/s00421-009-1303-3) 20012646 10.1007/s00421-009-1303-3

[2] Cardinale M. Bosco C.: ‘The use of vibration as an exercise intervention’, Exercise Sport Sci. Rev., 2003, 31, (1), pp. 3–7 (doi: 10.1097/00003677-200301000-00002) 10.1097/00003677-200301000-00002 12562163

[3] Schiessl H.: ‘Device for stimulating muscles’. EP97/04475, 1997

[4] Roger Talish C.R. McLeod K.: ‘Exercise equipment utilizing mechanical vibrational apparatus’. US20040067833 A1, 2004 https://www.google.com/patents/US20040067833

[5] Mischi M. Cardinale M.: ‘The effects of a 28 Hz vibration on arm muscle activity during isometric exercise’, Med. Sci. Sports Exercise, 2009, 41, (3), pp. 645–652 (doi: 10.1249/MSS.0b013e31818a8a69) 10.1249/MSS.0b013e31818a8a69 19204585

[6] Pujari A.N. Neilson R.D. Cardinale M.: ‘A novel vibration device for neuromuscular stimulation for sports and rehabilitation applications’. Conf. Proc. Annual Int. Conf. IEEE Engineering in Medicine Biology Society, January 2009, vol. 2009, pp. 839–844 10.1109/IEMBS.2009.5333675 19964248

[7] Pujari A.N.: ‘Development and evaluation of vibration apparatus and method for neuromuscular stimulation’. PhD thesis, University of Aberdeen, UK, 2016

[8] Couto B.P. Silva H.R. Barbosa M.P.: ‘Chronic effects of different frequencies of local vibrations’, Int. J. Sports Med., 2012, 33, (2), pp. 123–129 (doi: 10.1055/s-0031-1286294) 22187385 10.1055/s-0031-1286294

[9] Marín P.J. Rhea M.R. Marin P.J.: ‘Effects of vibration training on muscle strength: a meta-analysis’, J. Strength Cond. Res., 2010, 24, (2), pp. 548–556 (doi: 10.1519/JSC.0b013e3181c09d22) 20072044 10.1519/JSC.0b013e3181c09d22

[10] Griffin M.J.: ‘Minimum health and safety requirements for workers exposed to hand-transmitted vibration and whole-body vibration in the European union; a review’, Occup. Environ. Med., 2004, 61, (5), pp. 387–397 (doi: 10.1136/oem.2002.006304) 15090658 10.1136/oem.2002.006304 PMC1740769

